# Free Thyroxine (fT4) as a Potential Biomarker of Neurological and Functional Outcome in Acquired Brain Injury: A Prospective Multicenter Cohort Study

**DOI:** 10.3390/jcm12237433

**Published:** 2023-11-30

**Authors:** Chiara Mele, Sergio Bagnato, Antonio De Tanti, Lucia Francesca Lucca, Donatella Saviola, Laura Marcuccio, Pasquale Moretta, Federico Scarponi, Ernesto Losavio, Emilia Picciola, Valeria Pingue

**Affiliations:** 1 Department of Clinical-Surgical, Diagnostic and Pediatric Sciences, University of Pavia, 27100 Pavia, Italy; 2 Unit of Neurophysiology and Unit for Severe Acquired Brain Injuries, Rehabilitation Department, Giuseppe Giglio Foundation, 90015 Cefalù, Italy; sergiobagnato@gmail.com; 3 Cardinal Ferrari Centre, Santo Stefano Riabilitazione KOS-CARE, 43012 Fontanellato, Italy; antonio.detanti@centrocardinalferrari.it (A.D.T.); donatella.saviola@centrocardinalferrari.it (D.S.); 4 S. Anna Institute, 88900 Crotone, Italy; l.lucca@istitutosantanna.it; 5 Istituti Clinici Scientifici Maugeri IRCCS, Neurorehabilitation Unit of Telese Terme Institute, 82037 Telese Terme, Italy; laura.marcuccio@icsmaugeri.it (L.M.); pasquale.moretta@icsmaugeri.it (P.M.); 6 Department of Rehabilitation, San Giovanni Battista Hospital, 06034 Foligno, Italy; scarponifede@gmail.com; 7 Istituti Clinici Scientifici Maugeri IRCCS, Neurorehabilitation and Spinal Unit of Bari Institute, 70124 Bari, Italy; ernesto.losavio@icsmaugeri.it (E.L.); emilia.picciola@icsmaugeri.it (E.P.); 8 Istituti Clinici Scientifici Maugeri IRCCS, Neurorehabilitation and Spinal Unit of Pavia Institute, 27100 Pavia, Italy; valeria.pingue@icsmaugeri.it

**Keywords:** acquired brain injury, thyroid hormones, biomarker, rehabilitation, outcome

## Abstract

The potential involvement of thyroid hormones (THs) in the neurological and functional recovery of patients with brain damage has been hypothesized. We aimed at investigating the role of THs and their variations during the rehabilitation process as predictive biomarkers of neurological and functional outcome in patients with acquired brain injury (ABI). This prospective, multicenter cohort study included 220 patients with ABI consecutively admitted for a 6-month neurorehabilitation program. Data on the etiology of the brain injury, occurrence of seizures, neurosurgical procedures, and death during hospitalization were collected. Both at the baseline (T0) and at the end of the rehabilitation process (T1), the following variables were evaluated: thyroid function (TSH, fT4, and fT3) and outcome measure including the Glasgow Coma Scale (GCS), Glasgow Outcome Scale-Extended (GOS-E), and Functional Independence Measure (FIM) scale. During neurorehabilitation, a significant decrease in fT4 levels was documented in the population as a whole and in patients with severe ABI (*p* < 0.0001), whereas no significant variations were found in TSH and fT3 levels. No significant associations were found between THs and seizure occurrence, while the neurological and functional outcomes were associated with the variation in fT4 levels during rehabilitation. In particular, a higher magnitude of decrease in fT4 levels emerged as an independent predictor of more severe neurological damage (OR = 3.48, CI 95% 1.04–11.69, *p* = 0.04) and a lower functional recovery (β = −0.22, *p* = 0.01). In conclusion, serum fT4 variation during neurorehabilitation could represent a potential biomarker of neurological and functional outcome in patients with ABI. Further studies are needed to investigate the mechanisms underlying this association.

## 1. Introduction

Acquired brain injury (ABI) refers to both traumatic and nontraumatic brain injuries and continues to be one of the leading causes of long-term adult disability worldwide [1,2]. In 2005, neurological conditions, which mainly include neurological injuries and cerebrovascular disorders, represented 4.3% of the global burden of disease [3]. Severe ABI is associated with many physiopathological mechanisms that underlie heterogeneous clinical manifestations including deterioration in physical, cognitive, and emotional functions [4]. The long-term consequences of ABI potentially include neurological and functional alterations. Seizures are one of the most frequent neurological consequences of ABI [5,6], and their occurrence during neurorehabilitation represents a significant predictor of poor neurological and functional outcome [7].

The identification of early predictors of poor outcomes is a central issue in post-acute rehabilitation, and several researchers are trying to identify a reliable biomarker of disability after ABI, with controversial results. The potential involvement of thyroid hormones (THs) in the mechanisms related to neurological and rehabilitation outcomes has been hypothesized [8,9]. It is known that THs are essential for the development, regulation, and differentiation of neuronal cells and neuroglia, and hence for the homeostasis and function of the central nervous system (CNS) [10]. In addition, it has been demonstrated that THs have a key role in neurotransmission, mitochondrial regulation, and the function and modulation of GABAergic interneurons [11,12,13], which join microcircuits recruited during seizures [14]. Other functions of THs in the CNS include the regulation of neuronal plasticity, the stimulation of neurogenesis, as well as the modulation of the dynamics of the cytoskeleton and intracellular transport mechanisms [15]. These mechanisms overlap with those identified to promote the recovery of lost neurological functions during the first months after brain injury [15], thus suggesting a potential role of TH signaling in neurological and functional recovery after ABI.

In two previous retrospective studies conducted on two cohorts of patients with TBI and disorder of consciousness post-ABI, we found that TH parameters are associated with neurological and functional outcomes [9,16]. However, in the first study we did not evaluate the thyroid function during and at the end of the rehabilitation path, while in the second one thyroid function variables at discharge were available for only 50 subjects, and this hampered a full interpretation of the results.

The present study was designed to investigate the role of THs and their variations during the rehabilitation process as predictive biomarkers of neurological and functional outcome in a large prospective cohort of patients with ABI.

## 2. Materials and Methods

### 2.1. Study Design and Population

This observational, prospective, multicenter cohort study included 220 patients with ABI (109 subjects with traumatic brain injury (TBI) and 111 subjects with hemorrhagic stroke (HS) admitted to five Italian hospitals specialized in the neurorehabilitation of patients with acute brain injury between June 2021 and June 2022. The Coordinating Center was Istituti Clinici Scientifici (ICS) Maugeri of Pavia.

Eligibility criteria were (1) patients over the age of 18; (2) diagnosis of ABI (TBI or HS); (3) admission to a hospital emergency unit within 24 h after the event; (4) admission to a neurorehabilitation unit for a 6-month rehabilitation program. Exclusion criteria included the presence of known brain damage that occurred before the index date, previously known hypothalamic–pituitary disorders or neurological diseases, known thyroid dysfunctions, use of l-thyroxine or triiodothyronine (T3), and/or use of medications interfering with thyroid function.

Following the acute brain injury and post-acute hospitalization according to routine management, all patients underwent a 6-month inpatient rehabilitation program consisting of individual 3 h daily treatment cycles 6 days per week, which included a multidisciplinary approach: nursing and nutritional support, physiotherapy, occupational therapy, speech therapy, cognitive training, as well as social and neuropsychological assistance.

The study design conformed to the ethical guidelines of the Declaration of Helsinki and was approved by the local Ethical Committee of ICS Maugeri (#2503 CE). The participants’ authorized representatives signed a written informed consent.

### 2.2. Variables and Measurements

At the beginning of the study, according to the previously mentioned criteria, all participating hospitals collected the following information: age, sex, occurrence of seizures, etiology of brain injury, neurosurgical procedures, neurological and functional assessments, use and type of antiepileptic drugs (AEDs), and mortality during hospitalization.

Both on admission (T0) and at discharge (T1), the following variables were evaluated: thyroid function (TSH, fT4, and fT3) and outcome measure including Glasgow Coma Scale (GCS), Glasgow Outcome Scale-Extended (GOS-E) [17], and Functional Independence Measure (FIM) scale [18,19].

#### 2.2.1. Thyroid Function Test

Thyroid function tests (TSH, fT4, fT3) were performed on admission and at discharge from neurorehabilitation. Serum samples were assayed for TSH, fT4, and fT3 using an automated chemiluminescence assay system (Immulite 2000; DPC, Los Angeles, CA, USA). The primary method was a two-site, solid-phase chemiluminescent immunometric assay (TSH) or competitive immunoassay (fT4 and fT3). The reference ranges were 0.4–4.0 µIU/mL for TSH, 0.8–1.8 ng/dL for fT4, and 1.8–4.2 pg/mL for fT3.

#### 2.2.2. Seizures

In accordance with the International League Against Epilepsy (ILAE) criteria [20], seizures were classified into two categories based on the time elapsed from brain injury: ASS, if occurring 1–7 days after brain damage; US, if occurring >7 days after brain damage. If any paroxysmal event occurred during hospitalization, the patient underwent clinical evaluation and neurophysiology confirmation, with appropriate monitoring.

#### 2.2.3. Outcome Measures

The severity of brain injury was assessed according to the GCS, which represents a standardized system for evaluating the degree of neurological damage. GCS included three determinants: eye opening, verbal responses, and motor response or movement. These determinants were assessed according to a numerical value that indicated the level of consciousness and the degree of dysfunction. Total scores ranged from 15 to 3. Brain damage was classified as “mild” when the score was from 13 to 15. A score from 9 to 12 suggested a “moderate” brain injury, while a score equal to 8 or less indicated a “severe” brain injury [21].

Six-month outcomes were characterized using the GOS-E, a global 1–8 scale with the categories of death (score of 1), vegetative state (score of 2), lower severe disability (score of 3), upper severe disability (score of 4), lower moderate disability (score of 5), upper moderate disability (score of 6), and good recovery (lower and upper; scores of 7 and 8).

Functional outcomes were evaluated through the FIM scale, an 18-item measurement tool that investigates an individual’s physical, psychological, and social function [18,19]. The tool was used to assess the disability level as well as change in patient status in response to rehabilitation or medical intervention [22].

### 2.3. Statistical Analysis

Values are expressed as median and interquartile range (IQR), or absolute number and percentage (%) at the beginning and at the end of the study, and calculated as absolute variations over baseline values (Δ = T1–T0). Data were tested for normality of distribution by the Shapiro–Wilk test and log-transformed when needed, to correct for skewness. The Kruskal–Wallis test for continuous variables and the chi-square tests for categorical variables were used for comparisons between the subgroups of ABI severity (mild, moderate, and severe). Comparison between T0 and T1 was performed using the Wilcoxon test. Spearman’s correlation analysis was used to identify the association between thyroid function parameters and the clinical and functional characteristics of the population as a whole.

Univariate and multivariable logistic regression analyses were used to identify the independent predictors of seizure occurrence, neurological outcome, and mortality within 6 months of ABI. Odds ratio (OR), 95% confidence interval (95% CI), and related significant values obtained from regression are reported.

Multivariate linear regression analysis was conducted to test the potential predictive role of thyroid function parameter variation during rehabilitation for recovery and functional outcome. The multilinear models included combinations of independent variables encompassing age, sex, etiology and severity of ABI, neurosurgical procedures, seizures, use of AED, and thyroid function parameters. β coefficients and related significance values obtained from the models are reported. *p* < 0.05 was considered as statistically significant. Statistical analyses were performed using SPSS version 24 (Somers, NY, USA).

## 3. Results

### 3.1. Clinical and Functional Characteristics 

A summary of the clinical and functional characteristics of the whole population is reported in Table 1. The male-to-female ratio was 2:1, and the median age at diagnosis was 53 (IQR, 39–66) years. A hemorrhagic lesion was found in 111 patients (50.5%), whereas a TBI was detected in 109 patients (49.5%). As regards to HS, an intraparenchymal lesion was detected in 91 patients (82.0%) and an extraparenchymal lesion in 20 patients (18.0%). In the latter case, a subdural hematoma was found in 85.0% of cases. Concerning TBI, an intraparenchymal hemorrhage was found in 77 patients (70.6%), with a subdural hemorrhage in 45.9% of cases.

Neurosurgical intervention had been performed in 40.5% of patients and included craniectomy and vascular procedures.

During the observation period from acute care hospitalization to inpatient rehabilitation, seizure occurred in 38 subjects (17.3%). Overall, ASS were diagnosed in 16 cases (7.3%), US in 14 cases (6.4%), and 8 patients (3.6%) first presented ASS and then US. After ABI, prophylactic AED treatment was started in 91 subjects (41.4%), of whom 6 (6.6%) subsequently developed seizures.

Death during rehabilitative hospitalization was documented in 21 patients (9.5%).

According to the GCS assessed in the subacute phase, ABI was classified as severe in 40.9%, moderate in 29.1%, and mild in 30.0% of patients. Demographic characteristics and the type of lesion were comparable between the three classes of ABI severity, whereas patients with moderate ABI had a higher prevalence of US when compared with patients with mild and severe ABI (*p* < 0.01).

As expected, patients with severe ABI had a worst rehabilitation outcome (*p* < 0.0001) and a higher mortality rate (*p* < 0.01) than patients with mild-to-moderate ABI.

After the rehabilitation process, significantly improved FIM and GOS-E scores were documented both in the population as a whole and in the subgroups (*p* < 0.0001).

### 3.2. Post-ABI Thyroid Function

Thyroid function parameters evaluated at T0 and T1 are reported in Table 1. At baseline, TSH serum concentrations were below the reference range in 3.2% of cases, at the higher limit of the reference range or slightly increased in 9.2% of cases, and normal in the remaining 87.6%. All subjects with altered TSH concentrations had normal fT4 levels. Low fT3 levels were observed in two patients with low TSH levels. In subjects with normal TSH, fT4 and fT3 levels were low in 3.2% and 9.7% of cases, respectively. No correlation was found between TSH or fT4 levels and age, sex, or the etiology of ABI. Conversely, fT3 concentrations were lower in males (rho = 0.22, *p* < 0.01). While fT4 and fT3 levels were correlated with each other (rho = 0.28, *p* < 0.0001), no associations were found between TH and TSH levels.

During neurorehabilitation, a significant decrease in fT4 levels was found in the population as a whole and in patients with severe ABI (*p* < 0.0001), whereas no significant variations were found in TSH and fT3 levels.

Following the 6-month neurorehabilitation program, 7.3% of patients had low TSH levels, with low fT4 and fT3 levels in two cases. In patients with normal TSH or with TSH at the higher limit of the reference range, fT4 and fT3 levels were reduced in 3.3% and 6.6% of cases, respectively. TSH and fT4 levels did not correlate with age, sex, or the etiology of ABI, whereas fT3 levels decreased with age (rho = −0.34, *p* < 0.0001). No correlation was found between TSH, fT4, and fT3 levels.

No differences were found in thyroid function parameters between TBI and HS at baseline (T0) and after 6-month neurorehabilitation (T1).

### 3.3. Thyroid Function and Neurological/Functional Outcome

No associations were found between thyroid function parameters and seizures (ASS or US or both), independently of age, sex, ABI etiology and severity, neurosurgical procedures, and the use of AEDs. Concerning the use of prophylactic AEDs, there were no differences in thyroid function parameters between users and nonusers, nor when the three ABI groups were analyzed separately.

The neurological outcome in terms of GCS T1 was significantly associated with the variation in fT4 levels (ΔfT4) during rehabilitation. In particular, a higher magnitude of fT4 reduction during rehabilitation was associated with more severe neurological damage (OR = 3.48, CI 95% 1.04–11.69, *p* = 0.04), independently of sex, age, etiology of ABI, neurosurgical procedures, and seizures.

Bivariate correlation analysis was performed to evaluate the potential association between thyroid function parameters and rehabilitation outcome (Table 2). TSH and fT3 levels, as well as their variations, were not associated with recovery and functional outcome in terms of the GOS-E and FIM total score. Contrariwise, the fT4 level at discharge and its variations during rehabilitation were found to be strongly correlated with functional outcome in terms of the FIM total score (FIM T1: rho = −0.19, *p* = 0.03; ΔFIM: rho = −0.27, *p* = 0.002).

Multivariable linear regression models were built to evaluate the potential role of ΔfT4 as an independent predictor of functional outcome in terms of FIM variation (ΔFIM). The model that achieved the highest coefficient of determination (R2) is reported in Table 3. A higher magnitude of reduction in fT4 levels (ΔfT4) acted as a strong predictor of a lower variation in the FIM total score (ΔFIM), implying that greater decreases in fT4 during rehabilitation were associated with lower functional recovery (β = −0.22, *p* = 0.01). As expected, other predictors of rehabilitation outcome included GCS T0 (β = −0.37, *p* < 0.0001) and age (β = −0.23, *p* = 0.01).

### 3.4. Thyroid Function and Mortality

Mortality within 6 months of ABI was documented in 21 patients (9.5%). Univariate logistic regression analysis was performed to test the potential association between thyroid function parameters and mortality, and showed that lower fT3 levels were significantly associated with a higher mortality rate (OR = 0.06, CI 95% 0.01–0.40, *p* = 0.004). When the associative analysis was weighted for potential confounders in a multivariate logistic regression model, the association between fT3 levels and mortality was lost, while GCS T0 emerged as the only independent predictor of death within 6 months of ABI (Table 4).

## 4. Discussion

The present study investigated the potential association between thyroid function parameters and neurological and functional outcome in a large cohort of subjects with ABI. Our results did not show significant associations between THs and seizure occurrence, while the neurological and functional outcomes were associated with the variation in fT4 levels during rehabilitation. In particular, a higher magnitude of decrease in fT4 levels emerged as an independent predictor of more severe neurological damage and a lower functional recovery.

THs are crucial for adult brain function and have a key role in modulating neural stem cell function and neuronal plasticity after ABI [16,23,24,25]. Further, some authors hypothesized that THs could contribute to the pathogenesis of seizures [8]. In the context of ABI, the altered permeability of BBB could promote an abnormal entry of THs from the bloodstream into the brain, thus altering the mitochondrial function and promoting ROS generation and oxidative stress [26,27], which are associated with human epileptogenesis [28]. 

While in a previous study we found that fT3 levels were directly associated with an increased risk of US in TBI [9], in the present cohort of patients with ABI we did not find any association between THs and seizures. However, it is worth mentioning that in the previous study more than 65% of patients had a severe brain injury, with altered fT3 levels in half of the patients, whereas in the present cohort only 40% of patients had severe damage, with an alteration in fT3 levels in less than 10%. Therefore, the extent of the damage would appear to be higher in the previous population, and this could suggest a greater alteration in BBB permeability. The increased transcellular permeability of the BBB allows extravasation of immune cells, solutes, and proteins, as well as an abnormal entry of THs into the brain tissue, thus promoting abnormal neuronal excitability and epileptogenesis [29,30,31]. Overall, whether the TH-mediated mechanism could influence post-ABI epileptogenesis remains to be investigated in different settings and cohorts.

It is known that ABI represents an important cause of disability and death in adults [1,2]. The role of THs in neurological and functional outcome after ABI is still debated. THs are essential to ensure the proper functioning of the CNS [10]. Their functions are finely regulated by binding to specific membrane and intracellular receptors regulating non-genomic and genomic mechanisms in glial cells and neurons, respectively [15]. Moreover, THs modulate the processes of neuronal plasticity and intracellular transport, stimulate neurogenesis and angiogenesis, and regulate the dynamics of cytoskeletal elements. Based on this evidence, some authors have speculated on the potential role of THs in neurological and functional outcomes after ABI [15,16]. In our previous study on a large cohort of patients with a disorder of consciousness secondary to ABI, a wider variation in fT4 levels was associated with a worse functional disability level [32]. Our present results confirm that a higher magnitude of decrease in fT4 levels during rehabilitation represents an independent predictor of more severe neurological damage and a worse functional outcome. On the one hand, we could assume that higher variations in circulating fT4 reflect a condition of systemic imbalance, rather than acting as a predictor of neurological and functional recovery; on the other hand, specific mechanisms could be involved in this relationship. It has been demonstrated that in the secondary response to ABI, hypoxia leads to the activation of hypoxia-inducible factors (HIFs), increases the expression of the pro-inflammatory factor NF-kB, and releases NF-kB-mediated inducible nitric oxidase synthase (iNOS) [33]. Experimental studies on mice have suggested that iNOS has neuroprotective properties in brain injury [34,35]. TH concentrations are directly correlated with nitric oxide (NO) production in rat brain, and T4 induction of iNOS has also been observed in cortical neurons [33,36]. In particular, in post-injury rat cortex, T4 increases iNOS mRNA. Therefore, a higher decrease in fT4 levels during neurorehabilitation could be associated with a lower induction of iNOS in the brain, thus losing the neuroprotective effects of this molecule [37]. Studies have also demonstrated that TH signaling is fundamental to the proper functioning of short- and long-term synaptic plasticity [38,39], and a potential key role of T4 in the specific molecular patterns of neuronal plasticity processes has also been hypothesized [15]. Experimental studies showed that T4 can restore the polymerization of intracellular filaments [40,41] and promote polymerization of the actin cytoskeleton in neuronal and astrocyte cell cultures [42,43]. Also, basic transcription element-binding protein is upregulated by THs, and this protein could play a potential role in neuronal outgrowth, modulating cell differentiation [44,45]. In this setting, a higher magnitude of decrease in fT4 levels could affect neuroplasticity processes, thus compromising neurological and functional recovery. Several central and peripheral mechanisms could explain the intriguing association between fT4 concentrations and neurological/functional outcome, but in vitro and in vivo ad hoc studies are needed to explore this issue. 

From a clinical viewpoint, post-ABI variations in TH can act centrally and peripherally through modulation of neurotransmitters and neuronal homeostasis, as well as the induction of metabolic alterations in muscle and bone [16]. Therefore, we hypothesize that these two components may act synergistically to influence individual skills and rehabilitation outcomes. The evidence of an association between THs and neurological/functional outcomes led some researchers to investigate the use of TH replacement therapy to improve these outcomes, with controversial results [46,47].

Concerning mortality, it is known that ABI represents an important cause of mortality in adults [48,49]. In our previous study on patients with TBI, we observed that increasing TSH and declining fT3 levels were associated with a higher risk of death within 6 months following TBI [9]. In the present study, the univariate logistic regression analysis showed that lower fT3 levels were significantly associated with a higher mortality rate. Generally, a low fT3 level after injury or critical illness has been recognized as a strong predictor of systemic impairment and death [50]. However, after controlling for potential confounders, including age, sex, severity and etiology of ABI, neurosurgical procedures, and seizures, the severity of ABI emerged as the only independent predictor of death within 6 months of ABI.

Our study has some limitations. First, the study aimed to find associations, without insights into mechanisms, which need ad hoc investigations. Second, several methodological issues may have interfered with the laboratory tests used to asses thyroid function. Third, we did not assess the evolution of the thyroid function during the rehabilitation process, and this hampered a full interpretation of the observed associations.

In conclusion, serum fT4 variation during neurorehabilitation could represent a potential biomarker of neurological and functional outcome in patients with ABI. Further studies are needed to investigate the mechanisms underlying this association.

## Figures and Tables

**Table 1:** Clinical and functional characteristics of patients with ABI as a whole and subgrouped according to the Glasgow Coma Scale (GCS) on admission (T0).

Variables		Whole Population (n = 220)	ABI Severity (GCS T0)			
		N (%) Median (IQR)	Mild 66 (30.0)	Moderate 64 (29.1)	Severe 90 (40.9)	*p*-Value
Sex	Males	140 (63.6)	38 (57.6)	47 (73.4)	55 (61.1)	0.14
	Females	80 (36.4)	28 (42.4)	17 (26.6)	35 (38.9)	
Age (years) median (IQR)		53 (39–66)	51 (35–66)	54 (47–61)	53 (39–68)	0.90
Etiology of ABI	HS	111 (50.5)	34 (51.5)	32 (50.0)	45 (50.0)	0.98
	TBI	109 (49.5)	32 (48.5)	32 (50.0)	45 (50.0)	
Neurosurgical procedures	Yes	89 (40.5)	22 (33.3)	23 (35.9)	44 (48.9)	0.10
	No	131 (59.5)	44 (66.7)	41 (64.1)	46 (51.1)	
Seizures	Total	38 (17.3)	6 (9.1)	17 (26.6)	15 (16.7)	0.03
	ASS	16 (7.3)	3 (4.5)	4 (6.3)	9 (10.0)	0.39
	US	14 (6.4)	2 (3.0)	9 (14.0)	3 (3.3)	**<0.01**
	ASS + US	8 (3.6)	1 (1.5)	4 (6.3)	3 (3.3)	0.35
AED	No therapy	112 (50.9)	42 (63.76)	31 (48.5)	39 (43.3)	0.04
	Prophylactic	91 (41.4)	22 (33.3)	23 (35.9)	46 (51.1)	0.05
	For crisis	17 (7.7)	2 (3.0)	10 (15.6)	5 (5.6)	**<0.01**
TSH (µUI/mL)	T0	1.55 (0.94–2.41)	1.46 (0.98–1.99)	1.45 (0.83–2.23)	1.72 (0.99–2.73)	0.21
	T1	1.68 (1.07–2.55)	1.73 (0.92–2.36)	1.66 (1.15–2.87)	1.72 (1.08–2.66)	0.31
	Δ	0.07 (−0.40–0.71)	0.07 (−0.32–0.65)	0.19 (−0.17–0.85)	−0.07 (−0.57–0.46)	0.13
fT4 (ng/dL)	T0	**1.19 (1.02–1.49)**	1.17 (1.00–1.42)	1.14 (0.99–1.34)	**1.33 (1.08–1.59)**	**<0.01**
	T1	1.01 (0.85–1.21) *	1.04 (0.88–1.16)	0.93 (0.80–1.09)	1.07 (0.89–1.27) *	0.27
	Δ	−0.16 (−0.36–0.02)	−0.14 (−0.32–0.02)	−0.17 (−0.41–0.00)	−0.18 (−0.36–0.05)	0.23
fT3 (pg/mL)	T0	2.41 (2.10–2.98)	2.45 (2.17–2.92)	2.42 (2.09–2.85)	2.40 (2.03–3.00)	0.70
	T1	2.65 (2.16–3.05)	2.61 (2.30–3.00)	2.77 (2.26–3.13)	2.69 (2.09–3.09)	0.85
	Δ	0.08 (−0.37–0.69)	−0.01 (−0.38–0.48)	0.08 (−0.23–0.81)	0.20 (−0.46–0.72)	0.81
GOS-E	T0	**3 (2–3)**	**3 (3–3)**	**3 (2–3)**	**2 (2–3)**	**<0.0001**
	T1	3 (3–4) *	4 (3–5) *	3 (3–4)*	3 (2–3) *	**<0.0001**
	Δ	1 (0–1)	1 (0–1)	1 (0–1)	0 (0–1)	**<0.01**
FIM total score	T0	**18 (18–23)**	**26 (19–45)**	**18 (18–20)**	**18 (18–18)**	**<0.0001**
	T1	50 (22–93) *	85 (47–113) *	46 (25–90) *	25 (18–56) *	**<0.0001**
	Δ	27 (2–56)	46 (20–67)	27 (7–64)	4 (0–34)	**<0.0001**
Mortality	Yes	21 (9.5)	0 (0.0)	6 (9.4)	15 (16.7)	**<0.01**
	No	199 (90.5)	66 (100)	58 (90.6)	75 (83.3)	

Data are expressed as median and interquartile range (IQR) or absolute number and percentage. Comparisons between groups of ABI severity were performed with χ2 or Kruskal–Wallis tests. Comparison between T0 and T1 was performed using the Wilcoxon test; * *p* < 0.0001. Significant differences are shown in bold characters. Abbreviations: T0, on admission; T1, at discharge; HS, hemorrhagic stroke; TBI, traumatic brain injury; ASS, acute symptomatic seizures; US, unprovoked seizures; GOS-E, Glasgow Outcome Scale-Extended; FIM, Functional Independence Measure.

**Table 2:** Spearman’s correlation analysis to evaluate the association between thyroid function parameters and functional outcome.

Variables Rho Coefficient		TSH (µIU/mL)			fT4 (ng/dL)			fT3 (pg/mL)		
		T0	T1	Δ	T0	T1	Δ	T0	T1	Δ
GOS-E	T1	−0.05	0.02	0.05	−0.13	−0.11	−0.03	−0.06	0.04	0.02
	Δ	−0.02	0.03	0.002	−0.09	−0.002	0.06	−0.11	0.06	0.14
FIM	Tot T1	−0.03	0.01	0.07	0.01	−0.14	−0.19 *	0.02	0.03	−0.02
	Δ Tot	−0.02	0.02	0.04	0.11	−0.12	−0.27 **	−0.04	0.04	−0.01

Associative analysis was performed using Spearman’s correlation test; * *p* < 0.05, ** *p* < 0.01. Abbreviations: T0, on admission; T1, at discharge; TSH, thyroid-stimulating hormone; fT4, free thyroxin; fT3, free triiodothyronine; GOS-E, Glasgow Outcome Scale-Extended; DRS, Disability Rating Scale; FIM, Functional Independence Measure.

**Table 3:** Multivariable linear regression analysis showing independent predictors of ΔFIM.

	ΔFIM (R^2^ = 0.524)	
Model Independent Variables	Beta	*p*-Value
Age (years)	**−0.23**	**0.01**
Sex (M = 0, F = 1)	0.02	0.86
GCS T0 (Mild = 1, Moderate = 1, Severe = 3)	**−0.37**	**<0.0001**
Etiology of ABI (HS = 0, TBI = 1)	−0.12	0.22
Neurosurgical procedures (No = 0, Yes = 1)	−0.09	0.34
Seizures (No = 0, Yes = 1)	−0.01	0.93
Use of AED (No = 0, Yes = 1)	−0.09	0.45
ΔfT4 (ng/dL)	**−0.22**	**0.01**

Significant associations are shown in bold characters. Abbreviations: T0, on admission; FIM, Functional Independence Measure; GCS, Glasgow Coma Scale; HS, hemorrhagic stroke; TBI, traumatic brain injury; fT4, free thyroxine.

**Table 4:** Multivariable logistic regression models showing the potential risk factors for mortality within 6 months of brain injury in the population as a whole.

Covariates	Death within 6 Months of ABI (No = 0, Yes = 1)		
	OR	CI 95%	*p*-Value
TSH T0 (µIU/mL)	0.95	0.62–1.44	0.80
fT4 (ng/dL)	0.29	0.02–4.57	0.38
fT3 (pg/mL)	1.68	0.58–4.81	0.34
Age (years)	1.02	0.97–1.06	0.45
Sex (M = 0, F = 1)	0.61	0.14–2.61	0.51
GCS T0 (Mild = 1, Moderate = 1, Severe = 3)	**4.42**	**1.17–16.64**	**0.02**
Etiology of ABI (HS = 0, TBI = 1)	2.24	0.50–10.14	0.29
Neurosurgical procedures (No = 0, Yes = 1)	1.06	0.25–4.50	0.94
Seizures (No = 0, Yes = 1)	2.89	0.67–12.43	0.15

Significant associations are shown in bold characters. Abbreviations: T0, on admission; TSH, thyroid-stimulating hormone; fT4, free thyroxin; fT3, free triiodothyronine; BMI, body mass index; VS, vegetative state; MCS, minimally conscious state; TBI, traumatic brain injury.

## Data Availability

The data presented in this study are available on request from the corresponding author.
